# Characterization and Reliability of [^18^F]2FNQ1P in Cynomolgus Monkeys as a PET Radiotracer for Serotonin 5-HT_6_ Receptors

**DOI:** 10.3389/fphar.2017.00471

**Published:** 2017-07-18

**Authors:** Véronique Sgambato-Faure, Thierry Billard, Elise Météreau, Sandra Duperrier, Sylvain Fieux, Nicolas Costes, Léon Tremblay, Luc Zimmer

**Affiliations:** ^1^Université Claude Bernard Lyon I Lyon, France; ^2^Institut des Sciences Cognitives Marc Jeannerod, CNRS UMR5229 Bron, France; ^3^Institut de Chimie et de Biochimie, CNRS UMR5246 Villeurbanne, France; ^4^Centre de Recherche en Neurosciences de Lyon, CNRS UMR5292, INSERM U1028 Lyon, France; ^5^CERMEP-Imagerie du Vivant Lyon, France; ^6^Hospices Civils de Lyon Lyon, France

**Keywords:** PET imaging, serotonin, 5-HT_6_ receptor, striatum, non-human primate

## Abstract

Brain serotonin-6 receptor (5-HT_6_R) is the one of the most recently identified serotonin receptors. Accumulating evidence suggests that it is a potent therapeutic target for psychiatric and neurological diseases. Since [^18^F]2FNQ1P was recently proposed as the first fluorinated positron emission tomography (PET) radioligand for this receptor, the objective of the present study was to demonstrate its suitability for 5-HT_6_R neuroimaging in primates. [^18^F]2FNQ1P was characterized by *in vitro* autoradiography and *in vivo* PET imaging in cynomolgus monkeys. Following *in vivo* PET imaging, tracer binding indices were computed using the simplified reference tissue model and Logan graphical model, with cerebellum as reference region. The tracer binding reproducibility was assessed by test–retest in five animals. Finally, specificity was assessed by pre-injection of a 5-HT_6_R antagonist, SB258585. *In vitro,* results showed wide cerebral distribution of the tracer with specificity toward 5-HT_6_Rs as binding was effectively displaced by SB258585. *In vivo* brain penetration was good with reproducible distribution at cortical and subcortical levels. The automated method gave the best spatial normalization. The Logan graphical model showed the best tracer binding indices, giving the highest magnitude, lowest standard deviation and best reproducibility and robustness. Finally, 5-HT_6_R antagonist pre-injection significantly decreased [^18^F]2FNQ1P binding mainly in the striatum and sensorimotor cortex. Taken together, these preclinical results show that [^18^F]2FNQ1P is a good candidate to address 5-HT6 receptors in clinical studies.

## Introduction

Serotonin (5-hydroxytryptamine, 5-HT) is a central neurotransmitter involved in a wide variety of physiological functions, as well as in neurological and psychiatric disorders. Pharmacological studies identified numerous serotonergic receptor families and subtypes, classified by structural, functional and pharmacological criteria into seven distinct receptor classes (5-HT_1-7_) (Beaudoin-Gobert and Sgambato-Faure, 2014). Of these, the 5-HT_6_ receptor (5-HT_6_R) is a G protein-coupled receptor that has recently emerged as a new target for neuropsychopharmacology. The exclusive brain localization of this receptor (Woolley et al., 2004), its role at the interface of signaling pathways (Dawson, 2011; Dayer et al., 2015) and, finally, its presence in the pharmacological spectra of several psychotropic drugs have led to the suggestion that it may have applications in the treatment of various psychiatric disorders (Arnt and Olsen, 2011). More recently, it appeared that 5-HT_6_R is involved in memory (Meneses, 2014), cognitive processes (Benhamù et al., 2014) and regulation of food intake (Heal et al., 2011), reinforcing its status as an emerging target for anti-dementia and anti-obesity therapeutic agents. According to the United States and European trial databases, more than 20 clinical studies of 5-HT_6_R are currently open or have been recently completed, evidencing that 5-HT_6_R antagonists have a symptomatic efficacy profile that can be differentiated from that of currently used agents (cholinesterase inhibitors and the NMDA-antagonist, memantine) (Benhamù et al., 2014; Claeysen et al., 2015). Despite the fact that 5-HT_6_R functionality is much more complex than initially thought, it is now quite evident that this receptor has great pharmaceutical potential in terms of cognition enhancement.

Positron emission tomography (PET) is a powerful functional imaging modality using a radioligand (ligand coupled to radioactive isotope) that enables visualization and assessment of the distribution/density of a targeted receptor in a living subject (animal or human) (Lancelot and Zimmer, 2010; Zimmer and Luxen, 2012). PET is an important tool for elucidating the mechanisms underlying brain disease and enhancing basic and clinical research in neuropharmacology, particularly in Alzheimer’s disease (Bao et al., 2017). There is therefore a critical need for a specific PET radiopharmaceutical for molecular imaging of 5-HT_6_Rs.

5-HT_6_R was first visualized in rats, pigs and humans by *in vitro* autoradiography using 5-HT_6_R antagonists [^3^H]Ro 63-0563 and [^125^I]SB258585 (Boess et al., 1998; Sleight et al., 1998; Hirst et al., 2000; East et al., 2002; Hirst et al., 2003; Marazziti et al., 2013), with a finding of relative abundance of 5-HT_6_R-like immunoreactivity in extrapyramidal and limbic areas in rodents (Gérard et al., 1997; Roberts et al., 2002). More recently, regional distribution and associated neuronal or glial expression were investigated on human *post-mortem* tissue by both autoradiography with [^125^I]SB258585 and immunohistochemistry (Marazziti et al., 2012, 2013). Taken together, these cross-species studies, although excluding non-human primates, revealed high levels in the striatum, moderate levels in the hippocampus and cerebral cortex and low levels in the cerebellum. In PET imaging studies, only two 5-HT_6_R ligands have been successfully used *in vivo*: [^11^C]GSK215083, which binds to striatal 5-HT_6_Rs in pigs, baboons and humans but displays high affinity for 5-HT_2A_Rs essentially in the frontal cortex (Parker et al., 2012, 2015) and [^18^F]2FNQ1P, recently developed by our team, that binds with high affinity and specificity to 5-HT_6_Rs in cats and macaques and, interestingly, no affinity for 5-HT_2A_Rs, nor 5-HT_1B_Rs, 5-HT_2B_Rs and 5-HT_2C_Rs (Becker et al., 2014; Colomb et al., 2014).

Given the paucity of available information on the expression of 5-HT_6_Rs in cynomolgus monkeys and the potential transposition of the PET tracer [^18^F]2FNQ1P to humans for clinical investigation, the present study sought to characterize the precise distribution of [^18^F]2FNQ1P in cynomolgus monkeys at both cortical and subcortical levels. *In vitro* autoradiography demonstrated the cerebral distribution of the tracer and its specificity toward 5-HT_6_Rs. Following *in vivo* PET imaging, indices of tracer binding were computed using different models and methods of spatial normalization to identify the best method of analysis. To assess the reproducibility and specificity of the tracer, a test–retest reliability study was performed. Finally, the effects of pre-injection with the 5-HT_6_R antagonist SB258585 were investigated. All these experiments has the aim to demonstrate that the radiopharmacological profile of [^18^F]2FNQ1P is suited to studies in human subjects, as 5-HT_6_R exhibits a high degree of interspecies homology between macaques and humans (present data and Hirst et al., 2000; Marazziti et al., 2012, 2013; Becker et al., 2014).

## Materials and Methods

### Animals

Five healthy male cynomolgus monkeys (*Macaca fascicularis*) between 4 and 5 years old (young adults) and weighing between 3.4 and 7.4 kg were used. They were kept under standard conditions (12-h light cycles, 23°C and 50% humidity). Food and water were available *ad libitum*. All studies were carried out in accordance with European Communities Council Directive (2010/63/EU) as well as the recommendations of the French National Committee (2013/113) and the local animal ethics committee (CELYNE, C2EA-43). For the *in vitro* part of the study, following the “3Rs” rule (Reduction, Refinement, and Replacement) for animal experimentation, post-mortem tissue from previous studies was used (Beaudoin-Gobert et al., 2015; Sgambato-Faure et al., 2016).

### Drugs

SB258585 hydrochloride was obtained from Sigma–Aldrich (Saint Quentin Fallavier, France). For *in vivo* experiments, SB258585 was injected through a saphenous vein catheter at a concentration of 1.5 mg/kg, 10 min before the intravenous injection of the tracer (Becker et al., 2014).

### [^18^F]2FNQ1P Radiolabeling and Quality Controls

2FNQ1P precursor was synthesized in the institute of chemistry and biochemistry (Lyon, France) and the radiolabeling was made in the radiopharmacy unit of the CERMEP-imaging platform according to our recently published protocol (Becker et al., 2014; Colomb et al., 2014). Quality control consisted in determining radiochemical purity and specific activity by analytical HPLC assay of an aliquot of the radiolabeled product, with comparison to the calibration curve generated from solutions of known concentrations.

### *In Vitro* Autoradiography

*In vitro* autoradiography was performed on post-mortem tissue from three cynomolgus monkeys. Briefly, serial 50 μm-coronal sections from paraformaldehyde-fixed brain were mounted on glass slides, and allowed to air-dry before storage at -20°C until use. On the day of radiotracer synthesis, the slides were allowed to reach room temperature and were then incubated for 20 min in Tris phosphate-buffered saline (138 mM NaCl, 2.7 mM KCl, pH adjusted to 7.6) containing 37 kBq/mL (1 mCi/mL) [^18^F]2FNQ1P. For competition experiments, the slides were placed in the same buffer supplemented with SB258585 (the selective 5-HT_6_R antagonist) at a concentration of 10 nM. After incubation, slides were dipped in cold buffer (4°C), then in cold distilled water (4°C), and dried and placed on a phosphor imaging plate for 60 min (BAS-5000; Fujifilm).

### *In Vivo* Image Acquisition

Positron emission tomography and MRI acquisitions were performed in anesthetized *cynomolgus* monkeys [atropine at 0.05 mg/kg i.m., followed 15-min later by zoletil at 15 mg/kg i.m. as previously (Beaudoin-Gobert et al., 2015)].

Anatomical MRI acquisition comprised a 13-min 3D anatomic T1-weighted sequence using a 1.5-T Magnetom scanner (Siemens, MRI department of the CERMEP imaging platform). The MPRAGE sequence was used with the following acquisition parameters: TE = 2.89 ms, TR = 2160 ms, IT = 1100 ms, flip angle = 15°, FoV = 154 mm, matrix size = 256 × 256, number of acquisitions = 2. The anatomical volume covered the whole brain, with 176 planes of 0.6 mm cubic voxels.

Positron emission tomography imaging was performed using a Siemens Biograph mCT/S64 scanner (PET department of the CERMEP imaging platform) with spatial transverse resolution of 4.4 mm (Jakoby et al., 2011). Attenuation was obtained using a 1-min low-dose CT-scan, acquired before emission. Dynamic acquisition started with the i.v. injection of the radiotracer (mean dose ± SEM: 123 ± 11.9 MBq) and lasted 90 min. Biograph mCT/S64 emission images were reconstructed using the Siemens ultraHD PET algorithm with 12 iterations, 8 subsets and a zoom factor of 21. Reconstructed volumes were 109 slices (2.027 mm thickness, 256 × 256 matrices of 0.398 mm × 0.398 mm^2^ voxels), in a series of 28 sequential-frame series of increasing duration from 30 s to 5 min. Each monkey underwent two [^18^F]2FNQ1P injections (test and retest) separated by a period ranging from 2 to 13 weeks. In addition, three monkeys underwent an additional [^18^F]2FNQ1P acquisition [before (around 1 month) or after (between 1 and 3 months) the retest] to study the effects of a pre-injection of SB258585, a 5-HT_6_R antagonist.

### Image Processing

#### Regions of Interest Delineation

Region delineation used the *M. fascicularis* atlas implemented with the MAXPROB method (Ballanger et al., 2013). For data clarity, some regions were grouped together as follows: anterior cingulate cortex (ACC24/32 + ACC25); prefrontal cortex (dPFC + vPFC + mPFC); sensorimotor cortex (S1 + M1 + premotor cortex); parahippocampal and entorhinal cortices; temporal (superior + middle + inferior cortices); parietal cortex (superior + inferior parietal); anterior striatum (anterior caudate + anterior putamen + ventral striatum) and posterior striatum (posterior caudate + posterior putamen + ventral posterior putamen). For each individual animal, PET scans were summed over frames, before being registered between PET acquisitions to provide an averaged PET image. Two different methods of spatial normalization were used and compared: (i) a manual method in which the PET image is manually co-registrated on the *M. fascicularis* MRI template (affine transformation performed with four points located at various brain positions); (ii) an automatic method, in which the averaged PET image is automatically co-registered on the individual MRI (rigid transformation), and the individual MRI automatically co-registered on the MRI template (affine + non-linear transformations). Direct and inverse transformations were concatenated to directly resample template and atlases in the individual native spaces, or reciprocally normalize PET data to the template space.

#### Kinetic Modeling

Positron emission tomography studies were analyzed by suitable tracer kinetics modeling at regional and voxel-based level. The parameters computed were: non-displaceable binding potential (BP_ND_), using the simplified reference tissue model (SRTM) (Gunn et al., 1997) and the distribution volume ratio (DVR), using the Logan Reference model (Logan, 2000) calculated using frames from 0 to 90 min. As defined by Innis et al. (2007), *in vivo* BP_ND_ refers to the concentration ratio at equilibrium of specifically bound radioligand to that of non-displaceable radioligand in tissue. BP_ND_ is the typical measurement from reference tissue methods, as it compares the concentration of radioligand in receptor-rich to receptor-free regions. Compartmental modeling also established that BP_ND_ is equal to the *f*_NS_.*k*_on_.*B*_avail_/*k*_off_. So BP_ND_ is linked to the available receptor density and the affinity of the radioligand for its specific receptor. As previous data from Parker and collaborators (Parker et al., 2012, 2015) support the use of the cerebellum as a region of reference for high affinity 5-HT_6_R ligands, the cerebellum (excluding vermis) was used as the reference area for both models. Regional parametric values were obtained by modeling the mean regional kinetics, extracted in the native PET spaces with the MAXPROB method. Whole-brain parametric images were obtained by modeling kinetics at the voxel level. Parametric images were transformed to the common template space.

#### Reproducibility

The reliability of the BP indices derived from the SRTM and Logan Model (BP = DVR-1) was assessed by computation of three reproducibility indices from the test–retest measurements: (i) percentage change in mean (relative bias): percentage change calculated as the difference between test and retest values divided by the test value; this index includes random changes and systematic biologic error; (ii) typical error or within-subject SD of the bias, expressed as percentage of the mean: the typical error is calculated as the standard deviation of the differences between BP_ND_ test and BP_ND_ re-test, divided by the square root of the number of measurements; (iii) intra-class correlation coefficient (ICC) estimating respect of rank in a test–retest study: this depends on the size and the quality of the sample in the population: ICC = (MSBS - MSWS)/(MSBS + MSWS), where MSBS is the mean sum of squares between subjects, and MSWS is the mean sum of squares within subjects.

### Statistical Analyses

All statistical analyses were performed using STATA software version 10.1. For autoradiography, for which data were represented as mean ± SEM, a paired *t*-test was used to attest the effect of the antagonist on selected regions of interest. For PET imaging, the whole-brain analysis was adjusted using the Bonferroni correction according to the number of regions of interest included.

## Results

### [^18^F]2FNQ1P Radiosynthesis

Automated radiolabeling of 2FNQ1P leading to [^18^F]2FNQ1P was performed from its nitro-precursors at 150°C, with a radiochemical yield of 36% corrected for decay and 80–100 min radiosynthesis time (including HPLC purification and formulation). No radioactive by-products were observed and the HPLC conditions ensured good separation of the radiotracer from its respective nitro-precursor, as confirmed on quality control. Radiochemical purity was better than 98% and specific activity of [^18^F]2FNQ1P was between 100 and 150 GBq/μmol, corrected at EOS.

### [^18^F]2FNQ1P *In Vitro* Autoradiography

**FIGURE 1:**
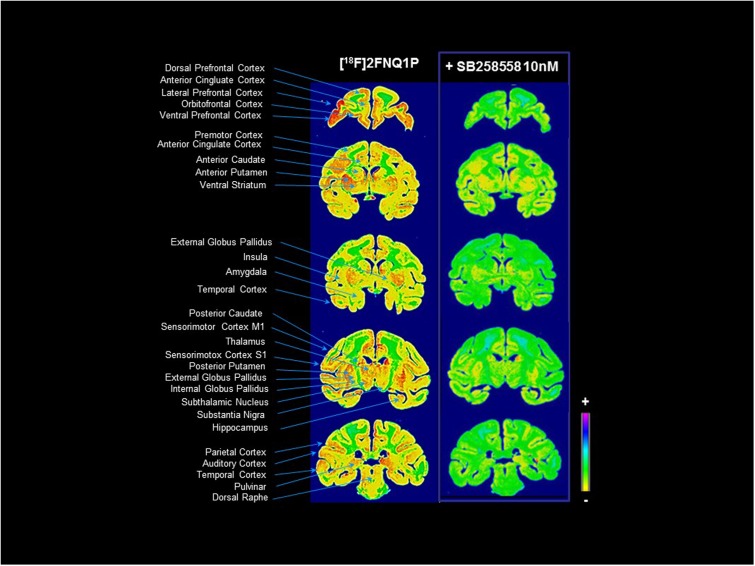
[^18^F]2FNQ1P BP_ND_ *in vitro* distribution in macaque brain. Monkey cerebral sections were processed for *in vitro* autoradiography with [^18^F]2FNQ1P under baseline condition and in presence of cold SB258585 (at 10 nM). Coronal sections were taken in the whole rostrocaudal extension of the monkey brain.

The *in vitro* autoradiography data evidenced a wide distribution of [^18^F]2FNQ1P in post-mortem tissue (**Figure 1**). The tracer bound to both cortical and subcortical areas, along the rostro-caudal axis of the brain. High binding levels were especially detected in the frontal cortex, anterior cingulate cortex, orbitofrontal cortex, basal ganglia, hippocampus and thalamus. Within the basal ganglia, high-to-moderate binding was detected in the striatum and pallidum, and in the subthalamic nucleus and substantia nigra. Lower levels of binding were observed in the insula, amygdala, raphe nuclei and periaqueductal region, parietal, occipital and temporal cortices, and the cerebellum. Interestingly, binding was substantially diminished in all regions after competition with SB258585 at a dose of 10 nM (**Figure 1**) (higher doses did not enhance this displacement; data not shown), demonstrating the sensitivity of [^18^F]2FNQ1P toward 5-HT_6_Rs, and encouraging *in vivo* investigation.

### [^18^F]2FNQ1P PET Imaging

**FIGURE 2:**
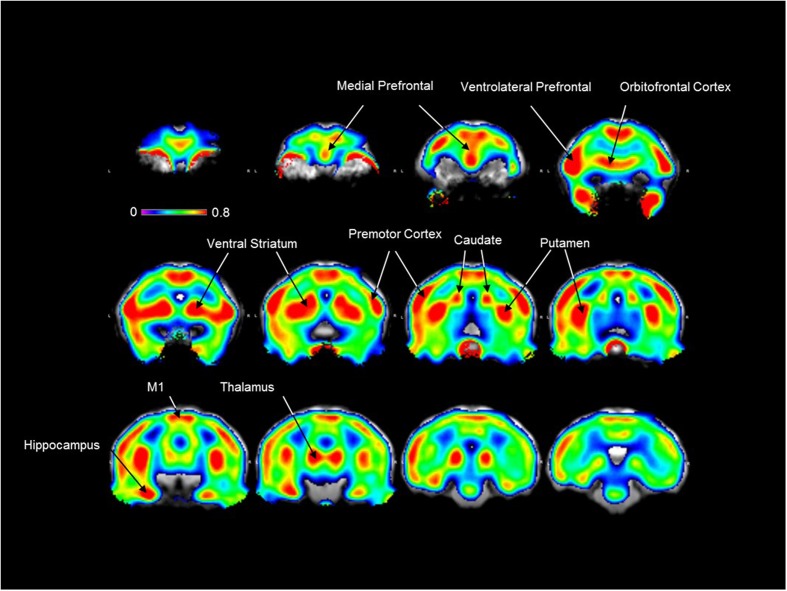
[^18^F]2FNQ1P BP_ND_ *in vivo* distribution in macaque brain. Spatially normalized parametric images of [^18^F]2FNQ1P BP_ND_ were averaged over five monkeys. The resulting mean image was superimposed on the *Macaca fascicularis* brain MRI template. Twelve coronal slices along the antero-caudal axis are shown. Colors represent the level of non-displaceable binding potential using the cerebellum as reference region, with the medial and ventrolateral prefrontal cortex, orbitofrontal cortex, premotor and motor cortices, ventral striatum, caudate nucleus, putamen, hippocampus and thalamus showing the highest binding values. Outside the brain, [^18^F]2FNQ1P binding is observed in the eyes and salivary glands but not in the skull.

As shown in **Figure 2**, a high [^18^F]2FNQ1P BP_ND_ was observed at baseline in primate brain regions, such as the prefrontal cortical areas, anterior cingulate cortex, striatum (ventral striatum, caudate nucleus, and putamen), thalamus, premotor and motor cortices. Additionally, moderate-to-low tracer fixation was detected in other cortical (parietal, temporal) and subcortical (amygdala, raphe nuclei) regions. Finally, binding is observed at the level of the eyes and salivary glands but not in the skull (**Figure 2**).

### Models and Methods of Spatial Normalization

Two models (SRTM and Logan) and two methods of spatial normalization (manual and automated) were compared, using three reproducibility indices: percentage change in mean, typical error and intra-class correlation coefficient (ICC). Combining all regions, change in mean progressively decreased from 35% (manual method and Logan model) to 6% (automated method and Logan model) (data not shown). Typical error diminished from 11% with the manual method to 5% with the automated method, independently of model (SRTM or Logan) (data not shown). Finally, ICC increased from 0.52 (manual method and SRTM model) to 0.58 (manual method and Logan model), reaching 0.69 with the automated method and SRTM model and 0.68 with the automated model and Logan model (data not shown).

### Test–Retest Reliability

**FIGURE 3:**
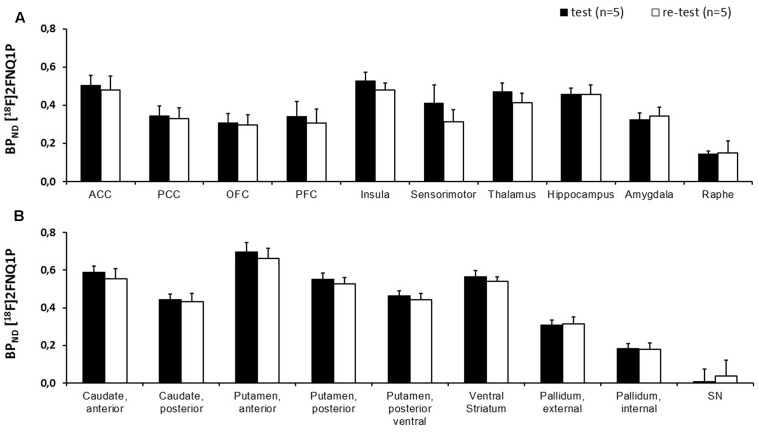
Comparison of BP_ND_ between test and retest scans. Histogram representing the BP_ND_ of [^18^F]2FNQ1P averaged over five subjects for test and retest scans in **(A)** neocortex and other areas and **(B)** basal ganglia. No significant difference between test and retest BP_ND_ was found on paired *t*-test with Bonferroni correction for multiple comparisons. Abbreviations: ACC, anterior cingulated cortex; PCC, posterior cingulated cortex; OFC, orbitofrontal cortex; PFC, prefrontal cortex.

Using manual spatial normalization and the SRTM model (data not shown), the BP_ND_ in test and retest ROIs went from 0.20 ± 0.14 in the PCC to 0.78 ± 0.05 in the anterior putamen. No difference was found between test and retest in the paired *t*-test comparisons. Mean percentage change by region went from -30% in the ACC to 148% in the PFC, with a range of typical error from 3% in the temporal cortex to 26% in the parietal cortex. ICC went from -0.17 in the amygdala to 0.83 in the temporal cortex. Using manual normalization and the Logan model (data not shown), BP_ND_ in test and retest ROIs went from 0.16 ± 0.10 in the internal pallidum to 0.76 ± 0.07 in the anterior putamen, with no significant difference. Mean percentage change by region went from -7% in the parahippocampal and entorhinal cortices to 150% in the internal pallidum, with a range of typical error from 7% in the ventral striatum and temporal cortex, to 18% in the PFC. ICC went from 0.16 in the amygdala to 0.86 in the ventral striatum. Using automated normalization with the SRTM model (data not shown), BP_ND_ in test and retest ROIs went from 0.13 ± 0.07 in the raphe to 0.67 ± 0.05 in the anterior putamen, with no significant difference. Mean percentage change by region went from -20% in the parahippocampal and entorhinal cortices to 43% in the raphe, with a range of typical error from 2% in the insula to 11% in the raphe. ICC went from 0.08 in the raphe to 0.97 in the PFC. Finally, using automated method and Logan model (**Table 1** and **Figure 3**), the BP_ND_ in test and retest ROIs went from 0.14 ± 0.02 in the raphe to 0.70 ± 0.05 in the anterior putamen, again with no significant difference. Mean percentage change by region went from -20% in the parahippocampal and entorhinal cortices to 27% in the raphe with a range of typical error from 2% in the temporal to 10% in the raphe. ICC ranged from 0.16 in the raphe to 0.97 in the prefrontal cortex. Of note, [^18^F]2FNQ1P exhibited BP_ND_ less than 1 as other serotonergic tracers ([^18^F]MPPF and [^11^C]GSK215083), which are used for *in vivo* imaging in both animals and humans studies (Yamamoto et al., 2013; Parker et al., 2015; Ballanger et al., 2016).

**Table 1:** Test–retest characteristics of BP_ND_ computed with automatic spatial registration and the Logan model.

Automatic LOGAN BP Region	Test (mean ± SEM)	Retest (mean ± SEM)	Change in mean (%)	Typical error (%)	ICC	Wilcoxon rank-sum test adjusted P
ACC	0.50 ± 0.05	0.48 ± 0.07	8.67	5.09	0.88	1.000
PCC	0.35 ± 0.05	0.33 ± 0.06	6.81	3.74	0.90	1.000
OFC	0.31 ± 0.05	0.30 ± 0.05	6.19	3.44	0.91	1.000
PFC	0.34 ± 0.08	0.31 ± 0.07	12.99	2.66	0.97	1.000
Insula	0.53 ± 0.05	0.48 ± 0.04	9.32	2.54	0.93	1.000
Sensorimotor	0.41 ± 0.10	0.31 ± 0.06	24.63	6.80	0.86	0.905
Temporal	0.25 ± 0.03	0.26 ± 0.02	-3.23	2.44	0.78	1.000
Parietal	0.35 ± 0.09	0.31 ± 0.07	11.35	6.33	0.88	1.000
**Mean Neocortex**	**0.38 ± 0.06**	**0.35 ± 0.06**	**9.59**	**4.13**	**0.89**	**0.99**
Caudate, anterior	0.59 ± 0.03	0.55 ± 0.05	7.86	4.78	0.77	1.000
Caudate, posterior	0.45 ± 0.03	0.43 ± 0.05	4.21	4.97	0.65	1.000
Putamen, anterior	0.70 ± 0.05	0.66 ± 0.05	5.68	4.05	0.88	1.000
Putamen, posterior	0.55 ± 0.03	0.53 ± 0.03	4.90	5.30	0.46	1.000
Putamen, posterior ventral	0.46 ± 0.03	0.44 ± 0.03	4.79	4.89	0.46	1.000
Ventral Striatum	0.57 ± 0.03	0.54 ± 0.03	4.53	4.77	0.45	1.000
Pallidum, external	0.31 ± 0.02	0.3 ± 0.041	0.72	6.01	0.26	1.000
Pallidum, internal	0.18 ± 0.03	0.18 ± 0.04	7.33	5.10	0.47	1.000
**Mean Basal Ganglia**	**0.48 ± 0.03**	**0.46 ± 0.04**	**5.00**	**4.98**	**0.55**	**1.00**
Thalamus	0.47 ± 0.05	0.41 ± 0.05	14.26	3.81	0.87	0.905
Amygdala	0.32 ± 0.04	0.34 ± 0.05	-3.97	2.90	0.90	1.000
Hippocampus	0.46 ± 0.03	0.45 ± 0.05	2.38	4.38	0.79	1.000
Parahipp/Entorhinal	0.23 ± 0.04	0.26 ± 0.03	-17.16	5.57	0.54	1.000
Raphe	0.14 ± 0.02	0.15 ± 0.06	26.83	9.60	0.16	1.000
**Mean Other areas**	**0.32 ± 0.03**	**0.32 ± 0.05**	**4.47**	**5.25**	**0.65**	**0.98**
**All regions**	**0.40 ± 0.04**	**0.38 ± 0.05**	**6.62**	**4.72**	**0.70**	**0.99**
Cerebellum	-0.01 ± 0.00	-0.01 ± 0.00	-12.92	0.29	0.90	1.000

*ACC includes ACC24/32 and ACC25. PFC includes dPFC, vPFC and mPFC. Sensorimotor includes S1, M1, and PMC. Parietal includes superior parietal, inferior parietal and precuneus. Anterior striatum includes anterior caudate, anterior putamen, and ventral striatum; posterior striatum includes posterior caudate, posterior putamen and ventral posterior putamen. Bold values correspond to the mean of the above cited sub-regions*.

### Pre-injection of 5-HT_6_R Antagonist SB258585

**FIGURE 4:**
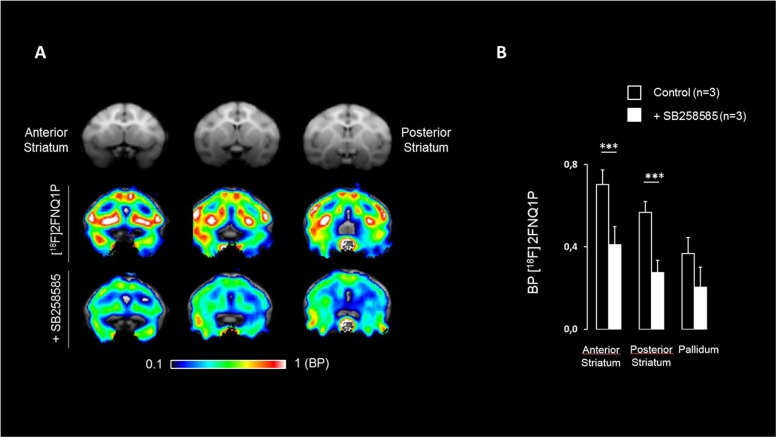
Effect of pre-injection of SB258585 on [^18^F]2FNQ1P BP_ND_. **(A)** *M. fascicularis* MRI template (first row), spatially normalized BP_ND_ image of one monkey at the baseline state (second row) and spatially normalized BP_ND_ image of the same monkey after antagonist administration (third row) are shown on three coronal slices along the striatum antero-caudal axis. Colors represent the level of binding using the cerebellum as reference region. **(B)** Histogram representing BP_ND_ of [^18^F]2FNQ1P, averaged over three monkeys, for the baseline state and after antagonist administration in the anterior and posterior striatum and the pallidum. It has to be noted that similar BP_ND_ decreases are measured in cortical regions (e.g., parietal, prefrontal, sensorimotor, and anterior cingulate cortices). Wilcowon rank-sum tests were used to compare baseline BP_ND_ with BP_ND_ after antagonist administration. ^∗∗∗^*p* < 0.003.

The specificity of [^18^F]2FNQ1P was tested by investigating the impact of pre-injection of SB258585 at a dose of 1.5 mg/kg, higher doses inducing cardiovascular manifestations such as breathing apnea in cat (Zimmer, personal communications). [^18^F]2FNQ1P BP_ND_ levels were significantly reduced in the striatum and sensorimotor cortex using a *p*-value threshold of 0.003 (i.e., 0.05/14 regions) to account for multiple comparison (**Table 2**). Decreases of [^18^F]2FNQ1P BP_ND_ were also observed in the ACC, PFC, Insula and parietal cortex with uncorrected *p*-value threshold (**Table 2**). These qualitative reductions of binding are illustrated in **Figure 4**. There were no correlation between [^18^F]2FNQ1P binding at baseline and the percentage of change in mean (*p* = 0.25, data not shown), evidencing that regions with high binding are not systematically the ones for which SB258585 displaced the more efficiently the radioligand.

**Table 2:** Effect of SB258585 pre-injection on BP_ND_ characteristics using manual and automatic spatial registrations with the Logan model.

Automatic LOGAN BP Region	Baseline (mean ± SEM)	Antago (mean ± SEM)	Change in mean (%)	SD change in mean (%)	Wilcoxonrank-sum test *P*-value
Anterior striatum	0.65 ± 0.10	0.45 ± 0.07	-28.85	13.13	0.0002***
Posterior striatum	0.54 ± 0.12	0.35 ± 0.07	-33.01	13.61	0.0002***
Pallidum	0.28 ± 0.08	0.30 ± 0.08	10.55	29.32	0.3668
ACC	0.49 ± 0.11	0.44 ± 0.09	-10.17	9.93	0.0076*
OFC	0.34 ± 0.11	0.27 ± 0.06	-18.89	21.76	0.1159
PFC	0.43 ± 0.18	0.38 ± 0.16	-9.96	17.69	0.0069*
Insula	0.57 ± 0.11	0.43 ± 0.07	-21.10	19.45	0.0464*
Sensorimotor	0.52 ± 0.20	0.30 ± 0.07	-37.52	16.80	0.0002***
Temporal	0.32 ± 0.16	0.33 ± 0.13	-2.58	42.73	0.3134
Parietal	0.48 ± 0.25	0.33 ± 0.08	-20.62	25.76	0.0414*
Thalamus	0.51 ± 0.10	0.46 ± 0.03	-8.04	16.04	0.2489
Amygdala	0.33 ± 0.10	0.34 ± 0.05	10.48	25.87	0.9165
Hippocampus	0.44 ± 0.10	0.42 ± 0.06	-3.73	9.88	0.3554
Cerebellum	-0.01 ± 0.05	-0.01 ± 0.07	-36.77	62.04	1.0000

*Baseline refers here to the mean of test and re-test BP_*ND*_. ACC includes ACC24/32 and ACC25. PFC includes dPFC, vPFC, and mPFC. Sensorimotor includes S1, M1, and PMC. Anterior striatum includes anterior caudate, anterior putamen and ventral striatum; posterior striatum includes posterior caudate, posterior putamen and ventral posterior putamen. Pallidum includes external and internal segments. ^∗∗∗^Indicates significant differences after Bonferroni correction for multiple comparison (i.e., *p*-value 0.003, *n* = 14). ^∗^Indicated significant difference (i.e., *p*-value < 0.05, uncorrected)*.

## Discussion

The serotonin system has been implicated in a variety of psychiatric and neurological disorders, and drugs targeting the 5-HT neurotransmitter system are commonly used to treat depression and anxiety. Serotonin 5-HT_6_R is one of the most recently discovered serotonin receptors almost exclusively localized in the brain (Woolley et al., 2004). 5-HT_6_Rs are increasingly reported to be involved in pathophysiological processes and possible therapeutics. Several pharmaceutical and biotech companies are currently developing new drugs specifically targeting this receptor: antipsychotics in schizophrenia, procognitive drugs in Alzheimer’s or Parkinson’s disease and dementia, and appetite suppressant drugs in obesity (Bali and Singh, 2015). There is therefore a critical need for a specific PET radiopharmaceutical suitable to the molecular imaging of this receptor. Ideally, a PET tracer is labeled with fluorine 18, which allow transfer to PET nuclear pharmacies and PET imaging centers, allowed thanks to its half-life (T½ = 110 min). Our recent data showed revealed that the 5-HT_6_R PET radiotracer our team developed, [^18^F]2FNQ1P, was the first to show satisfactory 5-HT_6_R selectivity and brain distributions consistent with known 5-HT_6_R density patterns in a feline model (Becker et al., 2014). Because 5-HT_6_R exhibits a high degree of interspecies homology between non-human primates and humans, and in order to reinforce the argument for transfer to clinical studies (first-in-man study), we characterized this radiotracer in cynomolgus monkeys, a non-human primate model classically used in translational studies in neuroscience. This test–retest reliability study of [^18^F]2FNQ1P binding was therefore designed to support interpretation of future preclinical and clinical studies, emphasizing the radiotracer binding pattern, reproducibility and precision of binding. The aim was to generate reliable and reproducible parameters to allow application in both cross-sectional and longitudinal studies.

Interestingly and encouragingly for translational purposes, the PET images were in agreement with the *in vitro* autoradiography data, confirming that [^18^F]2FNQ1P binds in a pattern exquisitely in accordance with cortical and subcortical 5-HT_6_R locations. Our *in vitro* results are in agreement with previous rodent and human studies showing higher 5-HT_6_ binding (and 5-HT_6_R mRNA) in the whole striatum compared to frontal and subcortical regions, and lower binding (and 5-HT_6_R mRNA) in the cerebellum (Roberts et al., 2002; Hirst et al., 2003; Marazziti et al., 2013). Further, the 5-HT_6_ selectivity of [^18^F]2FNQ1P’s *in vivo* binding was confirmed in the blocking study, in which striatal and cortical binding was decreased by a bolus of SB258585, a specific 5-HT_6_R antagonist, particularly in receptor-rich regions. Last and not least, this radioligand highly binds to cortical and subcortical regions, which are involved in behavioral disorders (Sgambato-Faure et al., 2016), strengthening the interest to study the potential therapeutic role of 5-HT_6_Rs in such disorders. Regions of potential interest for the use of this radiotracer are the striatum, thalamus and hippocampus, and the frontal, orbitofrontal, anterior cingulate, sensorimotor and insular cortices. The frontal cortex and hippocampus are regions critically involved in cognitive processing. However, the highest levels of 5-HT_6_Rs were found in motor and limbic regions of frontal cortex and striatum, which could indicate important roles of 5-HT_6_R in motor functions, motivation and reward processing (Tremblay et al., 2015; Sgambato-Faure et al., 2016).

Because spatial normalization is a critical step in the quantitative analysis of brain radiotracer binding (Matsuda, 2007), several models and methods were compared. In few words, several models are used to explore and quantify radiotracer kinetic in PET (Gunn et al., 1997; Logan, 2000; Innis et al., 2007). These models are formulated by differential equations ruling exchange between plasma and tissue compartments with the aim to simplify biological systems. Once a model is formulated, a tracer kinetic assay can provide very sensitive and accurate measurements of the rates of the process.

Our results clearly indicated that the automated method outperforms manual method. For BP computation, the Logan model showed slightly better results than the SRTM approach. We also investigated reproducibility indices separating splitting cerebral regions into neocortex, basal ganglia and other regions. Globally, reproducibility parameters were in favor of the automated normalization methods (higher ICC, lower typical error and bias) in cortical regions.

Compared to similar reproducibility studies published for other PET tracers of the serotoninergic system in primates (Chen et al., 2012; Tavares et al., 2014), the reproducibility parameters of [^18^F]2FNQ1P binding potential showed more satisfactory results. In consequence, the ability to detect changes in 5-HT_6_R availability or occupancy in *in vivo* studies, would be more sensitive with [^18^F]2FNQ1P, giving a supplemental chance of elucidating functional and dysfunctional serotonin transmission in the brain and facilitating the development of therapeutic drugs targeting 5-HT_6_Rs. Again, this sensitivity for detecting changes is a supplementary argument for transferring this radiotracer to human subjects.

However, several methodological points have to be taken into consideration. Firstly, possible modifications of the 5-HT_6_ receptor occupancy are limited by the robust methodology of the protocol: the same sex and age of the monkeys; the same scheduled time for each PET scans; the absence of any pharmacological treatment or other behavioral experiment during the test–retest period; the normalized anesthesia and monitoring procedures; the reproducible radiopharmaceutical characteristics of the radiotracer. Secondly, for a complete modeling of the [^18^F]2FNQ1P tracer kinetics, one should have the arterial input function of the compartment model. This arterial input function requires arterial blood samples, plasma centrifugation, and metabolites measurement. However, the metabolism of radiotracers is highly dependent on the P450 hepatic cytochromes and knowledge of the metabolite curve in primate experiments is poorly predictive of subsequent clinical studies (Burkina et al., 2017). Since arterial sampling is invasive and is not directly useful for future studies in human, we preferred therefore to restrict the current study to a test–retest and blocking study, which gave us enough knowledge about the *in vivo* characteristics of the tracer, while preserving an ethic reducing the intervention on the animal. This approach has already been followed for other serotonin PET tracers, e.g., [^18^F]Mefway, initially evaluated in the primate without arterial sampling (Saigal et al., 2006; Wooten et al., 2011) and later used in humans (Hillmer et al., 2014).

Taken together, the present results indicate that [^18^F]2FNQ1P is a reliable PET tracer for visualization and quantification of brain 5-HT_6_Rs in cynomolgus monkeys. Although these binding parameters were measured in non-human primates, we think that the radiopharmacological profile of [^18^F]2FNQ1P will be appropriate for studies in human subjects, as 5-HT_6_R exhibits a high degree of interspecies homology between non-human primates and humans. These data convinced us that a protocol can now be conducted in humans. This protocol will be similar with, in addition, the exploration of the nature of the radioactive metabolites of [^18^F]2FNQ1P and their lipophilicity, determining possible brain penetration. This first-in-man study will ultimately determine the utility of [^18^F]2FNQ1P as a PET ligand for 5-HT_6_R assessment in humans affected by several brain pathologies and/or treated with 5-HT_6_R drug candidates.

## Author Contributions

VS-F performed research, analyzed date and wrote the paper. TB performed research. EM analyzed data. SD performed research. SF performed research. NC analyzed data. LT wrote the paper. LZ designed the study and wrote the paper.

## Conflict of Interest Statement

The authors declare that the research was conducted in the absence of any commercial or financial relationships that could be construed as a potential conflict of interest.
